# Stratified analyses for selecting appropriate target patients with diabetic peripheral neuropathy for long-term treatment with an aldose reductase inhibitor, epalrestat

**DOI:** 10.1111/j.1464-5491.2008.02490.x

**Published:** 2008-07

**Authors:** N Hotta, R Kawamori, Y Atsumi, M Baba, H Kishikawa, J Nakamura, S Oikawa, N Yamada, H Yasuda, Y Shigeta

**Affiliations:** Chubu Rosai HospitalNagoya; * Department of Medicine, Metabolism and Endocrinology, Juntendo University School of MedicineTokyo; † Department of Internal Medicine, Saiseikai Central HospitalTokyo; ‡ Department of Neurology, Hirosaki University HospitalHirosaki; § Kumamoto University Health Care CenterKumamoto; ¶ Department of Endocrinology and Diabetes, Nagoya University Graduate School of MedicineNagoya; ** Division of Endocrinology and Metabolism, Department of Medicine, Nippon Medical SchoolTokyo; †† Department of Internal Medicine, Endocrinology and Metabolism, Graduate School of Comprehensive Human Sciences, University of TsukubaTsukuba; ‡‡ Department of Medicine, Shiga University of Medical Science, OtsuJapan; §§ Shiga University of Medical Science, OtsuJapan

**Keywords:** aldose reductase inhibitor, diabetic peripheral neuropathy, good condition of blood glucose level, polyol pathway

## Abstract

**Aims:**

The long-term efficacy of epalrestat, an aldose reductase inhibitor, in improving subjective symptoms and nerve function was comprehensively assessed to identify patients with diabetic peripheral neuropathy who responded to epalrestat treatment.

**Methods:**

Stratified analyses were conducted on data from patients in the Aldose Reductase Inhibitor—Diabetes Complications Trial (ADCT). The ADCT included patients with diabetic peripheral neuropathy, median motor nerve conduction velocity ≥ 40 m/s and with glycated haemoglobin (HbA_1c_) ≤ 9.0%. Longitudinal data on HbA_1c_ and subjective symptoms of the patients for 3 years were analysed (epalrestat *n* = 231, control subjects *n* = 273). Stratified analyses based on background variables (glycaemic control, grades of retinopathy or proteinuria) were performed to examine the relationship between subjective symptoms and nerve function. Multiple logistic regression analyses were conducted.

**Results:**

Stratified subgroup analyses revealed significantly better efficacy of epalrestat in patients with good glycaemic control and less severe diabetic complications. In the control group, no improvement in nerve function was seen regardless of whether symptomatic benefit was obtained. In the epalrestat group, nerve function deteriorated less or improved in patients whose symptoms improved. The odds ratio of the efficacy of epalrestat vs. control subjects was approximately 2 : 1 (4 : 1 in patients with HbA_1c_ ≤ 7.0%).

**Conclusion:**

Our results suggest that epalrestat, an aldose reductase inhibitor, will provide a clinically significant means of preventing and treating diabetic neuropathy if used in appropriate patients.

Diabet. Med. 25, 818–825 (2008)

## Introduction

Various drugs have been developed to treat diabetic neuropathy [1], including aldose reductase inhibitors (ARIs). ARIs suppress the activity of aldose reductase, a rate-limiting enzyme involved in the polyol pathway, which is enhanced in diabetic neuropathy. The effects of ARIs on diabetic neuropathy and diabetes-related complications have been investigated in animal and clinical studies [2,3]. Clinical efficacy of ARIs in diabetic neuropathy has been reported in terms of nerve function, subjective symptoms and histopathological findings of neural tissue [2–4]. Generally, parameters for nerve function, such as nerve conduction velocity and vibration perception threshold (VPT) are used as primary variables of efficacy of ARIs [5–9]. We previously reported the results of the 3-year Aldose Reductase Inhibitor—Diabetes Complications Trial (ADCT), which demonstrated the clinical efficacy of epalrestat, an ARI, in Japanese diabetic neuropathy patients with median motor nerve conduction velocity (MNCV) as the primary endpoint [10]. Stratified analyses of the ADCT data suggested that the effects of epalrestat on median MNCV were most evident in patients with better glycaemic control and without retinopathy or nephropathy [10].

Subjective symptoms may be more important to patients than nerve function. In ADCT, the efficacy of epalrestat was investigated by analysis of subjective symptoms such as numbness of upper and lower extremities, sensory abnormalities of lower extremities and cramp [10].

Here, we report additional analyses of ADCT data [10], in which stratified analyses of subjective symptoms were performed to identify patients likely to experience better responses to epalrestat. We determined the correlation between amelioration of subjective symptoms and nerve function to clarify the significance of ARIs in the treatment of neuropathy. Furthermore, we carried out logistic regression analysis using a comprehensive clinical assessment parameter based on nerve function and subjective symptoms and performed quantitative analysis of the efficacy of epalrestat adjusted for background variables.

## Patients and methods

ADCT was conducted at 112 medical facilities in Japan between 1997 and 2003. The protocol was approved by the Institutional Review Board of each medical facility and all patients gave informed consent.

The ADCT methodology has been reported previously [10]. Patients had mild diabetic peripheral neuropathy based on subjective symptoms, no foot ulcers and neurological dysfunction [at least two parameters: MNCV (indispensable) and VPT or Achilles tendon reflex, etc.]. Patients were enrolled if they had a median MNCV ≥ 40 m/s (seemingly reversible) and stable glycaemic control [glycated haemoglobin (HbA_1c_) ≤ 9.0%, with ± 0.5% variation in the previous 3 months]. Subjects were excluded if the primary cause of the neurological disorder was not diabetes (alcoholic neuropathy, carpal tunnel syndrome, sequelae of cerebrovascular disease, etc.), if they had peripheral arterial disease (ankle brachial pressure index of ≤ 0.8) or severe hepatic or renal disorder, if they were participating in other interventional studies, or if they were receiving other experimental medications for diabetic neuropathy, prostaglandin E_1_ preparations or any other medication that affects symptoms of diabetic neuropathy. Patients were randomized to either the epalrestat or control groups; details of the randomization method were described previously [10]. Epalrestat (50 mg) was administered orally three times daily before each meal (150 mg/day). Both groups continued conventional therapy (diet treatment, oral glucose-lowering agents, insulin and anti-hypertensive agents). With the exception of rescue medication, new medication to aid neuropathy control was prohibited.

### Study endpoints and measures of outcome

The primary endpoint was change from baseline to study end in median MNCV in the patient's non-dominant arm. This arm was chosen to avoid any bias as a result of possible lower limb impairment caused by the Japanese lifestyle (a tendency to sit straight). Secondary endpoints included changes from baseline to study end in minimum F-wave latency (MFWL) of the median motor nerve and VPT. VPT was measured using a 128-Hz tuning fork by measuring the time in seconds during which the patient felt vibrations. Changes of subjective symptoms of diabetic neuropathy were assessed using a 100-mm visual analogue scale (VAS). For a general measure of symptoms, the mean score was calculated for 10 symptoms (spontaneous pain in upper and lower extremities, numbness of upper and lower extremities, paraesthesia or hyperaesthesia of lower extremities, dizziness, cramp, coldness, abnormal sweating and constipation) and four symptoms in the lower extremities (spontaneous pain, numbness, paraesthesia or hyperaesthesia and cramp). The mean VAS of each symptom ranged from 20 to 30. The symptom score using in this study is the mean VAS of 10 symptoms per patient, although many values were zero. The mean symptom score at the beginning of the study in the control and epalrestat groups was 8.2 and 9.3, respectively.

The response to therapy was determined using a general assessment of subjective symptoms and nerve function. Patients were rated as responders if any of the following changes were observed over the 3-year study period: ≥ 1 m/s increase in median MNCV [11], ≥ 5% decrease in MFWL [12], ≥ 50% increase of time in VPT [13], or ≥ 50% decrease in the mean score for 10 symptoms [14].

### Statistical analysis

As for ADCT [10], efficacy analyses were performed in patients who had data for at least 1 year, using the last-observation-carried-forward (LOCF) method [15,16].

Statistical methods included χ^2^-tests for nominal scale, Mann–Whitney *U*-tests for ordered categorical scale, two-sample *t*-tests for comparison of mean values between groups and two-way repeated anova for changes in glycaemic control. Multiple logistic regression analysis was conducted using the defined parameters of efficacy. Normalization for the multiplicity of stratified analyses was not performed. All analyses were carried out using SAS Version 8.02 (SAS Institute, Cary, NC, USA). *P* < 0.05 was considered statistically significant.

## Results

### Patients

Patient clinical characteristics are provided in Table 1. There were no significant differences between the two groups.

**Table 1:** Patient characteristics at baseline

Patient characteristics	Control (*n* = 273)	Epalrestat (*n* = 231)	*P*-value
Sex			
Male	161 (59.0)	132 (57.1)	
Female	112 (41.0)	99 (42.9)	0.678*
Age (years)	61.5 ± 9.0	61.0 ± 10.0	0.541*
Duration of diabetes (years)	12.5 ± 8.0	13.2 ± 9.1	0.408*
Duration of neuropathy (years)	3.3 ± 3.6	3.7 ± 4.9	0.363*
HbA_1c_			
HbA_1c_ before and after treatment			
0 years	7.0 ± 0.1	7.2 ± 0.1	
3 years	7.2 ± 0.1	7.3 ± 0.1	0.122‡
Change over 3 years			
< 7.0%	71 (26.0)	51 (22.1)	
7.0% ≤ HbA_1c_ < 9.0%	156 (57.1)	141 (61.0)	0.470§
≥ 9.0%	46 (16.8)	39 (16.9)	

Data are means ± standard deviation (sd) or *n* (%).

*P*-values were calculated using

* χ^2^- tests

† two-sample *t*-test

‡ anova and

§ Mann–Whitney *U*-test. Duration of neuropathy refers to the mean patient-reported duration of neuropathy symptoms.

HbA_1c_, glycated haemoglobin.

In ADCT [10], the control and epalrestat groups included 305 and 289 patients, respectively. Of these, 31 and 55 patients withdrew after < 1 year, respectively; the reasons for withdrawal were a change in hospital (12 patients in each group), co-morbid illnesses (seven in each group), amelioration of symptoms (two epalrestat recipients), adverse events (20 epalrestat recipients), deterioration in symptoms (seven control subjects) or other (five control subjects, 14 epalrestat recipients). Both amelioration of symptoms and adverse events were observed only in the epalrestat group, resulting in a higher withdrawal rate in this group. Additionally, 59 and 53 patients had insufficient data for the primary efficacy analysis, primarily because of the unavailability of an electromyogram or a problem with the measuring technique. Thus, the primary efficacy analysis included 215 and 181 patients in the control and epalrestat groups, respectively.

This analysis included 273 patients in the control group and 231 patients in the epalrestat group, for whom data were available on symptom change and glycaemic control. The correlation between subjective symptoms and median MNCV was analysed in 214 patients in the control group and 179 patients in the epalrestat group.

### Changes in glycaemic control

The changes in HbA_1c_ observed in the two treatment groups are shown in Table 1. No significant differences between epalrestat recipients and control subjects in glycaemic control were observed at baseline or over 3 years of treatment.

### Stratified subgroup analyses of symptoms

**FIGURE 1:**
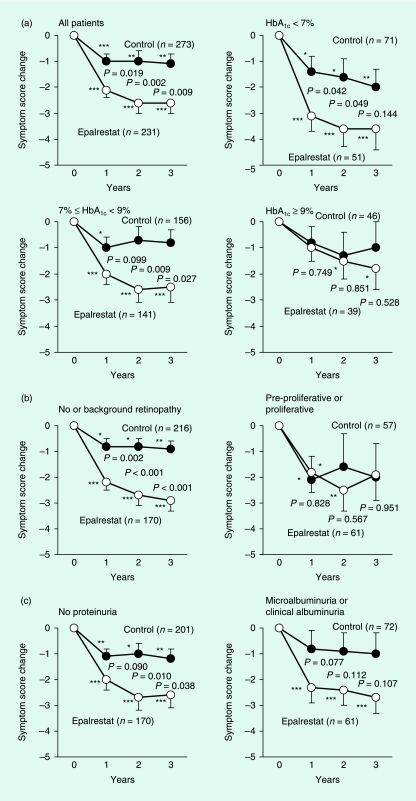
Effects of epalrestat on symptom score according to glycated haemoglobin (HbA_1c_) over 3 years (a), baseline level of retinopathy (b) and baseline level of proteinuria (c)., epalrestat group;control group. Data are reported as means ± standard error (se). *P*-values were calculated using the two-sample *t*-test. *P < 0.050, **P < 0.010 and ***P < 0.001 were calculated vs. baseline using the paired *t*-test. *P*-values are stated for between-group differences.

Stratified subgroup analyses were performed to examine the relationship between changes in symptom score for 10 symptoms and glycaemic control, grade of retinopathy and grade of proteinuria (Fig. 1).

In both groups, the mean symptom score improved significantly from baseline at years 1, 2 and 3 (Fig. 1a). The improvement was most evident in the epalrestat group and differences between the two groups were significant at years 1, 2 and 3 (*P* = 0.019, *P* = 0.002 and *P* = 0.009, respectively; Fig. 1a).

#### HbA_1c_

In patients with HbA_1c_ < 7.0%, the mean symptom score improved significantly in both groups at year 1, 2 and 3. The improvement was most evident with epalrestat and significant between-group differences were observed at years 1 and 2 (*P* = 0.042 and *P* = 0.049, respectively). In patients with HbA_1c_ ≥ 7.0% and < 9.0%, the control group showed significant improvement in the mean symptom score at year 1, whereas epalrestat recipients showed significant improvement at years 1, 2 and 3. Improvements in the mean symptom score were significantly greater with epalrestat than control at years 2 and 3 (*P* = 0.009 and *P* = 0.027, respectively). In patients with HbA_1c_ ≥ 9.0%, the control group showed no significant improvement in the mean symptom score at any time points, whereas the epalrestat group showed significant improvement at years 2 and 3. There were no significant between-group differences at any time points (Fig. 1a).

#### Grade of retinopathy

In patients with no or background retinopathy at baseline, the mean symptom score improved significantly in both groups at years 1, 2 and 3. The improvement was significantly greater in the epalrestat group at years 1, 2 and 3 (*P* = 0.002, *P* < 0.001 and *P* < 0.001, respectively). In patients with pre-proliferative or proliferative retinopathy, the control group showed significant improvement only at year 1, whereas the epalrestat group showed significant improvement at years 1 and 2; there were no significant between-group differences at any time points (Fig. 1b).

#### Grade of proteinuria

In patients with no proteinuria at baseline, the mean symptom score improved significantly in both groups at years 1, 2 and 3. The improvement was significantly greater with epalrestat at years 2 and 3 (*P* = 0.010 and *P* = 0.038, respectively). In patients who had microalbuminuria or clinical albuminuria at baseline, the epalrestat group showed significant improvement of the mean symptom score at years 1, 2 and 3, but the between-group differences were not statistically significant at any time points (Fig. 1c). The same trend was observed for four symptoms of the lower extremities (data not shown).

### Correlation between subjective symptoms and median MNCV

**FIGURE 2:**
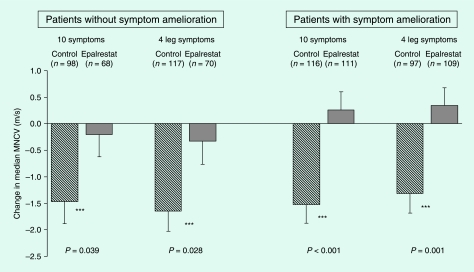
Amelioration of symptoms and change in median motor nerve conduction velocity (MNCV) after 3 years. Data are presented as mean ± se. *P*-values were calculated vs. baseline using a two-sample *t*-test for inter-group comparisons and a paired *t*-test for intra-group comparisons. 10 symptoms: spontaneous pain of upper and lower extremities, numbness of upper and lower extremities, leg paraesthesia or hyperaesthesia, dizziness, cramp, coldness, abnormal sweating, constipation; four leg symptoms: spontaneous pain, numbness, paraesthesia or hyperasthesia, cramp. ***P < 0.001 vs. baseline. *P*-values are stated for between-group differences.

Figure 2 shows changes from baseline in median MNCV according to symptom amelioration at year 3. In patients without improvement in the mean symptom score of 10 symptoms, deteriorations in median MNCV were –1.47 ± 0.41 m/s in the control group and –0.20 ± 0.42 m/s in the epalrestat group. Significantly less deterioration occurred in the epalrestat group (*P* = 0.039). In patients with improvement in the mean symptom score, median MNCV deteriorated by –1.52 ± 0.35 m/s in the control group and improved by 0.26 ± 0.34 m/s in the epalrestat group. Despite amelioration of symptoms, median MNCV deteriorated to a significantly greater extent in the control group than in the epalrestat group (*P* < 0.001). Furthermore, in the control group, median MNCV significantly deteriorated from baseline regardless of whether patients achieved symptom amelioration. As shown in Fig. 2, the same trend was observed for four symptoms of the lower extremities.

### Quantitative analysis of efficacy

**FIGURE 3:**
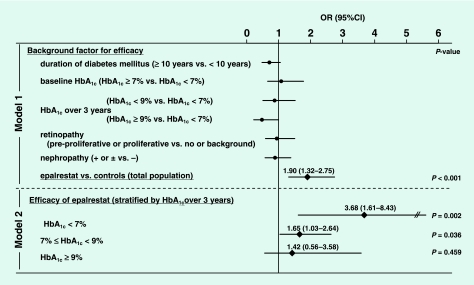
Logistic regression analysis analysing the efficacy of epalrestat vs. control. Efficacy analysis was based on a defined index, in which patients satisfying at least one of the following requirements during a 3-year period were classified as responders to treatment and odds ratios (ORs) were calculated: 1, ≥ 1 m/s increase in median motor nerve conduction velocity (MNCV); 2, ≥ 5% decrease in F-wave latency; 3, ≥ 50% increase of time in vibration perception threshold (VPT); 4, ≥ 50% decrease in the mean score of 10 symptoms.

The odds ratios (ORs) for achievement of a response to epalrestat therapy, based on analysis of nerve function and symptoms, are depicted in Fig. 3. In the multiple logistic regression model (Model 1), which was adjusted for the duration of diabetes mellitus, baseline HbA_1c_, HbA_1c_ over 3 years and grades of retinopathy and proteinuria, the OR for achievement of a response to epalrestat therapy was 1.90 [95% confidence interval (CI) 1.32–2.75, *P* < 0.001]. For Model 2, which was stratified by HbA_1c_ over 3 years, the OR of achieving a response to epalrestat therapy was 3.68 (95% CI 1.61–8.43, *P* = 0.002) for patients with HbA_1c_ < 7.0% and 1.65 (95% CI 1.03–2.64, *P* = 0.036) for those with HbA_1c_ < 9.0%. In patients with HbA_1c_ ≥ 9.0%, the OR was 1.42; this value was not significant vs. the control group.

### Safety

In ADCT, 26 of 295 subjects (the epalrestat group, including six patients of major protocol deviations) reported adverse events (AEs; 8.8%); these occurred within the first year of treatment in 22 of the 26 subjects. AEs included hepatic function abnormalities (seven cases), gastrointestinal symptoms such as nausea and diarrhoea (eight cases), skin rash/eczema (two cases) and one case each of vertigo, light-headedness, dorsal pruritus, hot flushes, hand stiffness, weakness of a lower extremity, oedema in a lower extremity, thirst and cerebral infarction. There were no severe AEs and no AEs were thought to be directly related to the long-term administration of epalrestat.

## Discussion

As it is not expected that cure of diabetes mellitus will be achieved, management of this condition focuses on maintaining long-term glycaemic control and reducing the occurrence and progression of diabetes-related complications, including diabetic neuropathy. If diabetic neuropathy symptoms deteriorate or persist, they can cause immeasurable mental and physical stress to patients [17]; serious complications such as ulcers or necrosis may culminate in amputation of an appendage [18]. Diabetic neuropathy is often associated with considerable mortality [19]. Therefore, it is of paramount importance to diagnose diabetic neuropathy immediately and provide an appropriate response to various risk factors [20,21] for the development and progression of diabetic neuropathy.

The first priority in the treatment of diabetic neuropathy is to maintain long-term glycaemic control. However, even when strict glycaemic control is achieved via traditional treatments, the occurrence and progression of diabetes-related complications remain unavoidable [22]. One important metabolic factor underlying diabetic neuropathy is an enhanced polyol pathway. Suppression of this pathway may be an important treatment strategy for diabetic neuropathy.

Diabetic neuropathy involves diverse symptoms such as spontaneous pain and numbness. Patients often complain of multiple symptoms. To determine distress in individual patients, numerous studies have calculated scores for multiple symptoms [14,23,24]. In this study, we conducted stratified analyses based on background variables, using the mean VAS score for 10 symptoms and the mean VAS score for four symptoms of lower extremities, with the aim of identifying patients expected to respond better to epalrestat therapy. The results indicated that achievement of symptom amelioration differed significantly between the epalrestat group and the control group for patients with HbA_1c_ < 7.0% or < 9.0%, with no or background retinopathy and no proteinuria. These results are consistent with those from stratified analyses on median MNCV reported previously [10] and provide evidence from both aspects of nerve function and subjective symptoms that epalrestat is more effective in patients with good glycaemic control and limited complications.

Moreover, in our analyses, patients continuing conventional therapy only (the control group) showed amelioration of symptoms and changes in glycaemic control, but with deterioration in median MNCV. This suggests that suppression of neuropathy progression is difficult to achieve with conventional therapy. In the epalrestat group, however, less deterioration or improvement in median MNCV were observed, regardless of whether patients achieved amelioration of symptoms. This suggests that epalrestat suppresses the enhanced polyol pathway, which is one of the important metabolic factors in the occurrence of diabetic neuropathy, and provides a useful option for treatment of diabetic neuropathy.

In diabetes mellitus, several reports have used multiple logistic regression analysis to determine treatment efficacy outcomes [25–27]. In this analysis, responders and non-responders to treatment were defined based on a general assessment of nerve function and subjective symptoms. Quantitative analysis of the efficacy of epalrestat, after adjustment for background variables, found that the OR was approximately 2 : 1 for the efficacy of epalrestat vs. control and 4 : 1 in patients with HbA_1c_ < 7.0%. The results of our comprehensive assessment of subjective symptoms and nerve function show clinically significant benefit of epalrestat treatment, particularly in patients with good glycaemic control. Although the parameters used in this analysis are of limited applicability, and careful interpretation is required, these data are consistent with previous results from the stratified analyses of median MNCV in ADCT [10], which were quantitatively endorsed.

The existence of bias cannot be ruled out because of the open-label trial design. However, measurement of nerve function by the medical technologist and the assessment of the electromyogram by the specialist physician were carried out under blinded conditions and amelioration and/or deterioration in subjective symptoms were correlated with changes in nerve function in patients treated with epalrestat. Therefore, although the overall design of this study may have some limitations, we consider this bias to have been minimized.

Suppression of an enhanced polyol pathway, which has an important role in the aetiology of diabetic neuropathy, cannot be ignored. Therefore, in addition to improving glycaemic control, treatment with ARIs is expected to play a significant role in the management of diabetic neuropathy. This comprehensive study suggests that epalrestat, an ARI, provides clinically significant benefit in preventing and treating diabetic neuropathy if used in the appropriate patients.

## Abbreviations

ADCT: Aldose Reductase Inhibitor—Diabetes Complications Trial
AEs: adverse events
ARI: aldose reductase inhibitor
HbA_1c_: glycated haemoglobin
MFWL: minimum F-wave latency
MNCV: motor nerve conduction velocity
VAS: visual analogue scale
VPT: vibration perception threshold
